# Vitamin D and Death by Sunshine

**DOI:** 10.3390/ijms14011964

**Published:** 2013-01-18

**Authors:** Katie M. Dixon, Wannit Tongkao-On, Vanessa B. Sequeira, Sally E. Carter, Eric J. Song, Mark S. Rybchyn, Clare Gordon-Thomson, Rebecca S. Mason

**Affiliations:** 1 Discipline of Physiology, Bosch Institute, School of Medical Sciences, University of Sydney, Sydney, NSW 2006, Australia; E-Mails: wton9162@uni.sydney.edu.au (W.T.-O.); vanessa@physiol.usyd.edu.au (V.B.S.); scar1097@uni.sydney.edu.au (S.E.C.); eson8487@uni.sydney.edu.au (E.J.S.); mrybchyn@mail.usyd.edu.au (M.S.R.); claregt@physiol.usyd.edu.au (C.G.-T.); rebecca.mason@sydney.edu.au (R.S.M.); 2 Discipline of Anatomy and Histology, Bosch Institute, School of Medical Sciences, University of Sydney, Sydney, NSW 2006, Australia

**Keywords:** vitamin D, 1,25-dihydroxyvitamin D3, ultraviolet radiation, sunburn cells, cyclobutane pyrimidine dimers, p53, nitric oxide, MAPK, AKT

## Abstract

Exposure to sunlight is the major cause of skin cancer. Ultraviolet radiation (UV) from the sun causes damage to DNA by direct absorption and can cause skin cell death. UV also causes production of reactive oxygen species that may interact with DNA to indirectly cause oxidative DNA damage. UV increases accumulation of p53 in skin cells, which upregulates repair genes but promotes death of irreparably damaged cells. A benefit of sunlight is vitamin D, which is formed following exposure of 7-dehydrocholesterol in skin cells to UV. The relatively inert vitamin D is metabolized to various biologically active compounds, including 1,25-dihydroxyvitamin D3. Therapeutic use of vitamin D compounds has proven beneficial in several cancer types, but more recently these compounds have been shown to prevent UV-induced cell death and DNA damage in human skin cells. Here, we discuss the effects of vitamin D compounds in skin cells that have been exposed to UV. Specifically, we examine the various signaling pathways involved in the vitamin D-induced protection of skin cells from UV.

## 1. Introduction

Ultraviolet radiation (UV) from the sun is well known to cause skin cell damage that ultimately leads to melanoma and non-melanoma skin cancer. Both the UVB (290–320 nm) and UVA (320–400 nm) wavelengths have been shown to contribute to the various molecular events that precede skin carcinogenesis [1,2]. The UVB component of UV is required for the production of vitamin D in skin cells. Exposure of 7-dehydrocholesterol in skin cells to UVB leads to production of pre-vitamin D3, which is then thermally isomerised to vitamin D3 [3]. This is then converted to the biologically active metabolite, 1,25-dihydroxyvitamin D3 (calcitriol; 1,25D) via two sequential hydroxylation steps, predominantly in the liver (forming 25-hydroxyvitamin D) and kidneys (forming 1,25D). There is now clear evidence that other cell types including skin cells are capable of producing 25-hydroxyvitamin D and 1,25D [4,5]. Continued exposure of pre-vitamin D and vitamin D to UV radiation produces additional photoproducts, including lumisterol [3].

The potentially harmful effects of UV radiation on skin cells include DNA damage and the production of reactive oxygen species (ROS) that interact with DNA and other molecules causing oxidative damage [6]. Nitric oxide (NO) levels are also increased by UV upregulation of nitric oxide synthases [7–9] and by UVA-photodecomposition of pre-existing NO stores [10–12]. NO loses its beneficial effects at high levels and acts as a free radical or combines with ROS to produce more toxic NO derivatives, such as peroxynitrite, which induce oxidative and nitrosative damage to DNA, nitrosylation of tyrosine residues in proteins, and initiate lipid peroxidation [13–15]. Peroxynitrite also activates poly(ADP-ribose) polymerase that converts NAD^+^ to nicotinamide and ADP-ribose. This reduces NAD^+^ and ATP formation resulting in energy depletion that leads to cell death [13,16].

An immediate deleterious effect of UV radiation is the production of various forms of DNA damage in skin cells. DNA damage and signal transduction pathways that are activated in response to stress related protein kinases activate a DNA damage response that leads to cell cycle arrest. This permits removal of DNA lesions by DNA repair enzymes before transcription and replication, or removal of irreparably damaged cells by apoptosis [1,17]. However, excess levels of NO cause inactivation of DNA repair enzymes by nitrosylation [18] and inhibit the excision and ligation steps of nucleotide excision repair [19]. NO overproduction can also alter the membrane potential of mitochondria, which facilitates the release of pro-apoptotic proteins such as apoptosis initiating factor [20]. Irreparable DNA damage and imbalances in antioxidant enzyme systems and scavengers are factors responsible for the induction of pro-apoptotic signaling pathways that lead to cell death.

## 2. Vitamin D and Cell Death

Vitamin D and analogs have been reported to induce apoptosis in several cancers, including but not limited to, prostate, breast, and colon cancers [21–23]. More recently, vitamin D compounds have been implicated in protection from skin cell death following UV exposure. The active vitamin D hormone 1,25-dihydroxyvitamin D_3_ (1,25D) was shown to reduce UV-induced cell death in cultured human skin cells [24–31]. The concentrations of 1,25D and doses of UV used in these studies vary considerably as noted in Table 1, although the spectra of the UV sources has not always been provided, making comparisons difficult. Studies by our group have demonstrated protection against UV-induced cell death with concentrations of 1,25D as low as 0.01 nM–10 nM in skin fibroblasts [28] and 1 nM–100 nM in keratinocytes [29] using 200 mJ/cm^2^ UVB and 1170 mJ/cm^2^ UVA. Others have achieved protection against UV-induced keratinocyte death using concentrations of 1,25D within this range [31] and at the upper end of this range [30], while another group has reported the need for much higher concentrations of 1,25D to inhibit UV-induced apoptosis, up to 1 μM [26]. Interestingly, the same group did not observe any cytotoxicity with this high dose of 1,25D, while other groups have reported cytotoxicity with doses of 1 μM [31,32]. A similar protective effect has been observed using vitamin D analogs; UV-induced skin cell death was inhibited by calcipotriol [33], 1α,25(OH)_2_lumisterol_3_ (JN) and 1α,25(OH)_2_-7-dehydrocholesterol (JM) [28] and 1α-hydroxymethyl-16-ene-24,24-difluoro-25-hydroxy-26,27-bis-homovitamin D_3_ (QW) [34].

**Figure 1:**
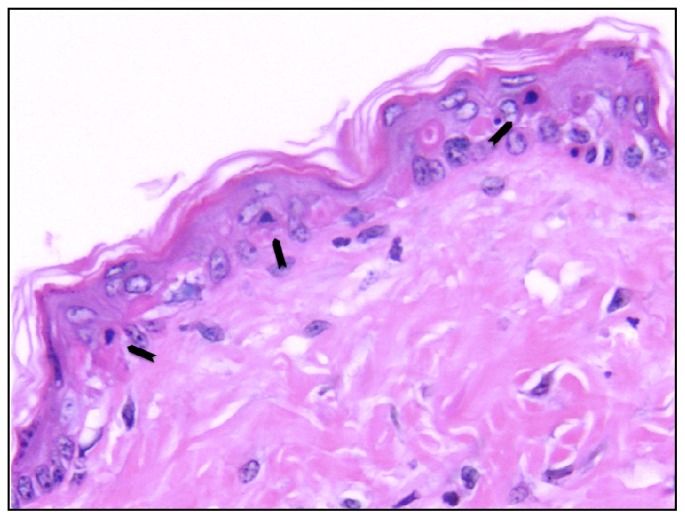
Typical histology of Skh:hr1 mouse skin showing “sunburn cells” or apoptotic keratinocytes, as indicated by black arrows. Sunburn cells are recognised by their pyknotic nucleus and eosinophilic cytoplasm.

Studies in mice and human subjects have also demonstrated a protective role for vitamin D compounds in preventing skin cell death. Apoptotic keratinocytes are known as “sunburn cells”, recognizable by their haemotoxylin-stained pyknotic nuclei and eosinophilic cytoplasm [35] (Figure 1), and are likely eliminated from skin before replication [36]. Sunburn cells were reduced in UV-irradiated mouse skin by 1,25D treatment, both systemically [37] and by topical application [29,38,39], and in UV-irradiated skin of human subjects after topical application of 1,25D [40].

## 3. UV-Induced Skin Cell Death: Mechanisms of Photoprotection by Vitamin D Compounds

One or more physiological responses observed in UV irradiated skin after vitamin D treatment may well contribute to the improved cell survival due to vitamin D. These responses include a reduction in UV-induced DNA damage [29,41,42], a reduction in nitric oxide metabolites, nitrite, 3-nitrotyrosine and 8-nitroguanosine [29,39,43,44], the upregulation of p53 [39,45] and the upregulation of inherent antioxidant systems, such as metallothionein [31,37]. These responses are discussed in more detail below.

### 3.1. Vitamin D Reduces UV-Induced DNA Damage

One of the major reasons for skin cell death following UV exposure is that the cells have acquired irreparable DNA damage. The most abundant forms of DNA damage found in UV irradiated human skin are thymine dimers, a subset of cyclobutane pyrimidine dimers (CPDs) formed by UV absorption [46–48], and 8-hydroxy-2′deoxyguanosine (8-oxodG) produced indirectly by reactive free radicals generated by UV [6,13,49,50]. Although a reduction in skin cell apoptosis after UV might allow more DNA-damaged cells to survive, the data show that both types of DNA photolesions are reduced in irradiated skin cells treated with 1,25D. This was demonstrated by immunohistochemistry using monoclonal antisera specific for thymine dimers and 8-oxo-dG. Image analysis showed reduced nuclear staining for thymine dimers in UV-irradiated skin cells treated with 1,25D in culture [28,38,51,52], in mouse skin [29,39,52], *ex vivo* human skin [43] and human skin *in vivo* [40]. Reduced thymine dimers in UV-exposed keratinocytes in the presence of 1,25D was also reported using an entirely different method, the Comet assay, which uses a specific enzyme to cut DNA at the site of the relevant lesion [44,53]. Reduced nuclear staining for UV-induced 8-oxo-dG by 1,25D was observed in mouse skin [44] and *ex vivo* human skin [43]. A reduction in 8-oxo-dG in UV irradiated keratinocytes by 1,25D has also been shown by Comet assay [44]. UV-induced strand breaks identified by a specific repair enzyme for 8-oxo-dG (human 8-oxoguanine DNA glycosylase) were reduced by 1,25D in irradiated keratinocytes in culture [44]. The reduction in thymine dimers and 8-oxo-dG occurred within 30 min after irradiation in culture [29,44], and also in human *ex vivo* skin when tested at three and six hours, post UV [43]. The reduction in UV-induced DNA damage would not only enhance cell survival, but is also likely to reduce some of the other deleterious effects of UV in human skin.

There is some information on mediators of this protective response. Vitamin D receptor knock-out mice show increased susceptibility to photocarcinogenesis [54]. At least in human skin fibroblasts, the 1,25D-induced reduction in UV-induced thymine dimers required the presence of a vitamin D receptor and of a stress protein ERp57 [45]. Interestingly, although a vitamin D receptor was required, mutations in either the DNA-binding domain or the classical ligand-binding domain, which produced the syndrome of vitamin D-resistant rickets in the donors, did not abrogate the protection from UV-induced thymine dimers in the presence of 1,25D [45]. This suggests that 1,25D may be acting via a non-classical, non-genomic pathway in exerting its photoprotective effects on DNA damage. This hypothesis is further supported by studies with analogs, including 1,25dihydroxylumisterol, which mimic, at least to some extent, the protective effect of 1,25D and by data which shows that the reduction in thymine dimers by 1,25D is lost in the presence of a chloride channel blocker 4,40-diisothiocyanatostilbene-2,20-disulfonic acid (DIDS) [53], shown to block non-classical effects of 1,25D in other cell types [55].

### 3.2. Effects of Vitamin D on p53

UV exposure leads to accumulation of p53 protein within the nucleus of skin cells. A well-known tumor suppressor, p53 is known as the “guardian of the genome” in that it may disrupt the cell cycle, allowing time for repair of DNA damage before replication [56]. Alternatively, following its phosphorylation at Ser46, p53 may stimulate apoptosis of cells with irreparably damaged DNA before replication.

Studies by our group have shown further increases in p53 at three and six hours after UV in keratinocytes and melanocytes treated with 1,25D (1 or 10 nM) [29,52] or vitamin D analogs [34,39]. Conversely, studies by another group have shown that at much higher concentrations, 1,25D (1 μM) suppresses p53 in UV-irradiated human keratinocytes measured at two, four and eight hours after UV [51]. The UVB doses used in these studies were also different. De Haes *et al.* postulated in the latter study that the reduction in p53 accumulation by 1,25D is in accordance with its ability to reduce DNA damage in the form of CPDs [51]. A more recent study by Yamaguchi and colleagues [57] demonstrated an increase in p53 paralleled by a reduction in CPDs in human subjects who had been exposed to repeated doses of UV. While this group attributed the protective response to an increase in pigmentation, the production of 1,25D and other D compounds in skin with repeated UV exposure may also contribute.

Nucleotide excision repair (NER) is the main mechanism for repair of UV-induced DNA damage. Interestingly, in keratinocytes, 1,25D increased the levels of two of the key enzymes involved in NER; XPC (xeroderma pigmentosum complementation group C) and DDB2 (damage-specific DNA binding protein 2 also known as XPE) [58]. Moreover, increased p53 also increases the levels of these enzymes [59] and, as stated above, 1,25D causes a further increase in expression of p53 after UV [29,39]. Whilst the 1,25D-mediated increase in p53 would presumably facilitate DNA repair, the proposed association between higher p53 and greater DNA repair has been challenged by studies that reported a dissociation between increases in p53 and levels of post-UV DNA damage in keratinocytes [53]. Furthermore, studies in dermal fibroblasts have shown that the increase in p53 by 1,25D can occur in the absence of the VDR, although in the total absence of VDR, no photoprotection by 1,25D is seen in these cells [45].

### 3.3. Vitamin D Compounds Reduce Nitric Oxide Derivatives

As noted earlier, UV increases NO in skin [7–9,11,12,60] leading to: oxidative and nitrative modifications to DNA and other molecules [15], inhibition of DNA repair [18,19], energy depletion and cell death [13,16], and membrane alterations; changes that permit the release of pro-apoptotic proteins from mitochondria and the induction of apoptosis.

There is evidence to suggest that 1,25D diminishes the incidence of oxidative and nitrosative DNA damage by reducing the production of NO and other toxic RNS, which may well improve DNA repair mechanisms and improve cell survival. Two relatively stable end products of the nitric oxide pathway, nitrite and 3-nitrotyrosine, used as measures for NO production, were significantly reduced in UV-irradiated skin cells in the presence of 1,25D when measured by the Griess assay (for nitrite) or a whole cell ELISA using a nitrotyrosine antibody [29,39]. Another nitric oxide product examined was 8-nitroguanosine (8-NG), which is a marker for inflammation and carcinogenesis [61]. UV-irradiated human *ex vivo* skin showed markedly increased level of 8-NG, and this was significantly reduced when treated with 1,25D in human *ex vivo* skin as early as 30 minutes post irradiation [43]. Similarly, nitric oxide synthase (NOS) inhibitors, such as aminoguanidine and L-*N*-monomethylarginine, reduced nitrite and thymine dimer production in irradiated cells to an extent comparable with that resulting from 1,25D treatment [28,29]. The selective inhibitor (1400W) of inducible NOS reduced CPDs and 8-oxo-dG in irradiated keratinocytes in culture [44].

### 3.4. Vitamin D and Antioxidant Systems

Activation of photoreceptors in skin by UV absorption generates free radicals by electron transfer or hydrogen abstraction processes in other molecules, or by energy transfer to molecular oxygen increasing the rate of ROS production [6]. During normal cellular metabolism low levels of ROS are regulated by intrinsic antioxidant enzyme systems and scavengers that maintain the redox balance in cells. For example, superoxide anions are converted by superoxide dismutase to hydrogen peroxide, which is converted to water and oxygen by catalase. Insufficient enzyme activity increases the levels of superoxide and hydrogen peroxide that form highly toxic peroxynitrite and hydroxyl ions respectively. Free radical scavengers, glutathione, metallothionein, thioredoxin, vitamin C and E, and carotenoids are also present in skin, but may be inactivated by UV-induced ROS and RNS. Glutathione peroxidase, superoxide dismutase and catalase expression are downregulated for several days following UV exposure, which would permit an exponential increase in ROS and reactive nitrogen species (RNS) [62]. Inflammatory cells induced by UV also migrate into irradiated skin and may contribute to increased levels of ROS [13]. An imbalance between ROS and antioxidant systems by UV will perturb cellular defence against DNA damage, oxidative and nitrative modifications to proteins, lipid peroxidation of membranes, leading to cell death.

Treatment with 1,25D inhibited the activation of stress-activated protein kinases, c-Jun *N*-terminal kinase (JNK), which lead to apoptosis in UV irradiated keratinocytes [26], and inhibited apoptosis induced by oxidative stress, TNFα and hydrogen peroxide cytotoxicity [25,63,64], possibly by increasing inherent antioxidant systems. Metallothionein, a cysteine-rich protein responsible for metal detoxification and an oxygen radical scavenger, when upregulated by cadmium in irradiated mouse skin, reduced UV-induced apoptotic sunburn cells, cell death and photo-damage [65], and was also shown to reduce superoxide and hydroxyl radicals [66]. UV-induced immune suppression was increased in metallothionein knockout transgenic mice, providing further evidence of its photoprotective effect [67,68]. Treatment with 1,25(OH)_2_D_3_ upregulated the transcription of metallothionein [69] along with a reduction in UV-induced sunburn cells in skin [31,37].

### 3.5. Vitamin D and MAPK Signaling

The mitogen activated protein kinase (MAPK) cascade is a large group of conserved protein serine/threonine kinases which consist of three distinct tiers of protein kinases; a mitogen-activated extracellular signal-regulated kinase kinase kinase (MEKK), which activates a mitogen-activated extracellular signal-regulated kinase kinase (MEK) that subsequently activates a MAPK. MAPKs include the well characterised c-jun-*N*-terminal kinase (JNK), extracellular signal-regulated kinase (ERK), and p38 MAPK. These kinases are widely located in keratinocytes and are targeted by various extracellular stimuli, including UVR [70]. Activation of MAPKs requires dual phosphorylation of specific residues within the activation loop, however skin cells must be exposed to UVR for a sufficient time, dose, and particular wavelengths for this to occur [71]. Upon activation, these members are able to phosphorylate each other in a series of well-defined cascades [70].

UVR-activated JNK and p38 MAPK translocate to the nucleus of the cell to initiate transcription of target genes resulting in the modulation of cellular functions [26,70]. JNK and p38 MAPK have been described in a number of studies as being involved in the induction of apoptosis [71]. However, treatment of keratinocytes with high concentrations of 1,25D (1 μM) [26] or at more physiological concentrations (1 nM) [72] leads to inhibition of UVR-induced JNK phosphorylation by 30%–50%. Since JNK promotes programmed cell death, this effect may contribute to the anti-apoptotic role of 1,25D in keratinocytes following UVR. Conversely, 1,25D has no effect on p38 MAPK activation following UVR exposure [26].

Treatment of keratinocytes with the JNK signaling inhibitor, SP600125, showed a significant decrease in UVR-induced cell loss, which was similar to the level of protection after 1,25D treatment [72]. When used in isolation, SP600125 inhibits phosphorylation of downstream targets of JNK but does not affect the phosphorylation of JNK itself [73].

### 3.6. Vitamin D and Akt Signaling

Similar to the raf/MEK/ERK (MEK/ERK) pathway, the phosphatidylinositol 3-kinase (PI3K)/Akt pathway is also constitutively active at a low level in epidermal keratinocytes and both ERK and Akt are upregulated by UVR [74,75]. Both these cascades play a key role in regulation of epidermal cell proliferation and cellular survival in response to extracellular stimuli [76]. Interestingly, it was reported that phosphorylation of ERK and Akt are augmented from basal levels in the presence of 1,25D which leads to modification of the expression and activity of various apoptosis-regulating molecules, which was suggested to decrease the susceptibility of keratinocytes to apoptosis [77]. Treatment of cells with pharmacological inhibitors of ERK and PI3K partly reverses protection against UVR-induced apoptosis by 1,25D, thus suggesting that the MEK/ERK and PI3K/Akt cascades may contribute to 1,25D-mediated cellular survival in UV-exposed human keratinocytes [77]. However, it should be noted that in the report by De Haes *et al.*, high concentrations of 1,25D (10 nM–1 μM) were required for this effect to be observed. Our group has reported a significant reduction in UV-induced thymine dimers in keratinocytes treated with 0.022 μM ERK peptide inhibitor or 0.2 nM Wortmannin, similar to that of 1,25D (1 nM) [78]. In at least some systems, Akt phosphorylation can result in increased beta-catenin phosphorylation and translocation to the nucleus [79]. There are several reported interactions between the vitamin D pathway and beta-catenin in skin [80]. In this context it is interesting to note that, in preliminary experiments, an inhibitor of β-catenin, IWR-1-endo, which enhances proteasomal degradation of β-catenin, on its own, also reduced thymine dimers after UV (unpublished observations). These results suggest that these pathways may contribute to UV-induced thymine dimers and presumably also UV-induced apoptosis, but further studies are required to test this. Protection against thymine dimers also remained when cells were treated with the combination of 1,25D and the ERK peptide inhibitor, Wortmannin [78] or IWR-1-endo (unpublished observations).

## 4. Conclusions

**Figure 2:**
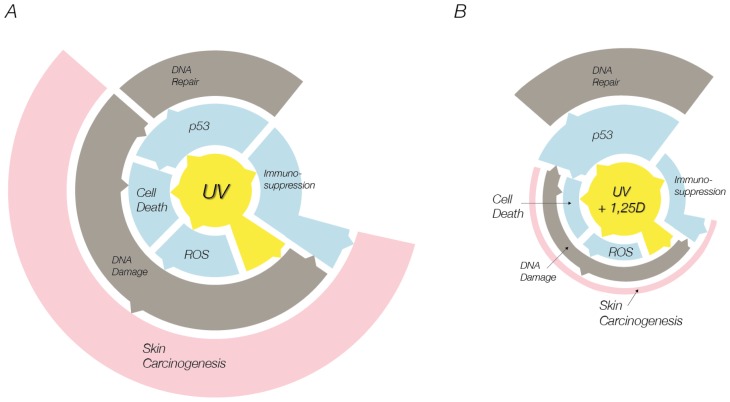
(**A**) Mechanisms of skin carcinogenesis due to ultraviolet (UV) radiation. UV causes cell death through p53-dependent and -independent mechanisms. p53 may facilitate DNA repair or can induce apoptosis of irreparably damaged cells. UV also causes DNA damage directly via absorption and indirectly via formation of reactive oxygen species. UV and DNA damage can lead to immunosuppression. Together, DNA damage and immunosuppression can initiate skin carcinogenesis; (**B**) Proposed mechanism for protection against skin carcinogenesis by 1,25D. The sizes of segments indicate reductions and increases caused by 1,25D. Vitamin D compounds can inhibit UV-induced cell death, DNA damage, immunosuppression and skin carcinogenesis. This may involve the ability of vitamin D compounds to further increase p53 after UV, thereby facilitating DNA repair.

Taken together, the studies reviewed here indicate that the inhibition of UV-induced cell death by vitamin D compounds is indeed a protective effect. This is evidenced by the further increase in p53, which facilitates DNA repair, as demonstrated by a reduction in CPDs in UV-irradiated skin cells treated with vitamin D compounds. Moreover, the indirect DNA damage and the reduction in DNA repair that is normally caused by nitric oxide products would be avoided by the ability of vitamin D compounds to reduce nitric oxide products. Vitamin D compounds are known to inhibit UV-induced immunosuppression [38,39,52], and a reduction in DNA damage would presumably result in inhibition of UV-induced immunosuppression [81]. Figure 2 shows a schematic of the proposed mechanism for protection against skin carcinogenesis by vitamin D compounds.

It could be argued that a reduction in UV-induced skin cell apoptosis in the presence of vitamin D metabolites might allow more damaged cells to survive and thus increase photocarcinogenesis. In practice however, it seems likely that the reduction in UV-induced apoptosis by vitamin D compounds is mainly a result of the reductions in UV-induced DNA damage observed in the presence of vitamin D compounds. This, together with reduced UV-immunosuppression, almost certainly underlies the observed reduction in skin carcinogenesis when vitamin D metabolites are used topically after UV irradiation [39]. Therefore, there is a potential role for vitamin D compounds as preventative agents for skin cancer.

## Figures and Tables

**Table 1:** Analysis of various studies of protection against UV-induced cell death by 1,25D, taking into account the UV output, dose of 1,25D and skin cell type.

References	UVB (mJ/cm^2^)	UVA (mJ/cm^2^)	1,25D Dose (nm)	Cell type
Gupta *et al*. 2007 *J. Invest. Derm*. ([29])	200	1173	1–100	Keratinocytes
Wong *et al*. 2004 *J. Ster. Biochem. Mol. Biol*. ([28])	200	1173	0.01–10	Fibroblasts
Lee & Youn 1998 *J. Derm. Sci*. ([31])	50	-	1.2 & 12	Keratinocytes
Manggau *et al*. 2001 *J. Invest. Derm*. ([30])	11.76	-	100	Keratinocytes
De Haes *et al*. 2003 *J. Cell. Biochem*. ([26])	32	-	100–1000	Keratinocytes
